# Application and Mechanisms of Biochar in Anaerobic Digestion: Towards Process Resilience and Waste Valorization

**DOI:** 10.3390/toxics14090764

**Published:** 2026-08-26

**Authors:** Yuan Shi, Yin Luo, Tingting Zhu, Kaijia Zang

**Affiliations:** 1 Faculty of Digital-Intelligent Urban Construction & Creative Design, Wenhua College, Wuhan 430074, China; 2 School of Environmental Science and Engineering, Tianjin University, Tianjin 300072, China

**Keywords:** biochar, anaerobic digestion, direct interspecies electron transfer (DIET), abiotic–biotic coupling, resource valorization

## Abstract

Anaerobic digestion (AD) is widely used for organic waste stabilization and bio-energy recovery, but its performance is often constrained by process instability, slow syntrophic metabolism, and sensitivity to acidification, ammonia, organic overloading, and inhibitory contaminants. Biochar has emerged as a promising strategy to enhance AD, with benefits extending beyond increased methane yield. This review examines how biochar properties, including pore structure, surface functional groups, alkalinity, and electrical conductivity, regulate different AD stages and improve process stability. Biochar provides microbial habitats, buffers pH, adsorbs inhibitors, accelerates volatile fatty acid conversion, and facilitates interspecies electron transfer. The effects of biochar dosage, feedstock type, reactor configuration, and operational conditions on digestion performance and resource recovery are critically evaluated. Broader contributions to organic waste valorization are also discussed. Future research should prioritize tailored biochar design, standardized characterization, long-term validation in continuous reactors, techno-economic analysis, and life-cycle assessment. These efforts are essential for translating laboratory findings into reliable and sustainable applications for organic solid waste treatment and resource recovery.

## 1. Introduction

The escalating global production of organic waste, including kitchen waste, agricultural residues, livestock manure and municipal sludge, has currently placed growing pressure on existing waste management systems [1]. The surge in regional footprints is notably evidenced by the increase in European Union (EU)-generated organic waste from 98 million tons in 2018 to 221 million tons in 2023. Concurrently, China reported a dramatic fourfold increase, from 14 million tons in 2018 to 60 million tons in 2025. Despite its huge generation volume, more than 70% of this massive organic waste is still landfilled, openly dumped, or incinerated with limited energy or nutrient recovery. Such practices not only waste biodegradable carbon and nutrients but also cause greenhouse gas emissions, odor release, and secondary environmental risks. Under the increasing demand for climate mitigation, renewable energy production, and circular economy development, organic waste treatment is no longer expected to merely achieve volume reduction and stabilization. Driven by the escalating energy crisis and the “dual carbon” strategy, the treatment of organic solid waste has shifted from conventional volume reduction toward resource recovery.

Anaerobic digestion (AD) is a promising biological technology for organic waste stabilization and bio-energy recovery (e.g., CH_4_, H_2_ and medium-chain fatty acids). Through four sequential stages, hydrolysis, acidogenesis, acetogenesis and methanogenesis, complex organic matter can be converted into methane-rich biogas and nutrient-containing digestate. However, AD performance depends on the metabolic synchrony among hydrolytic bacteria, fermentative bacteria, syntrophic acetogens and methanogenic archaea. This coordination is easily disrupted by organic overloading, volatile fatty acid (VFA) accumulation, ammonia inhibition and toxic compounds. Recent metagenomic evidence further shows that overload-induced instability is closely associated with the blockage of butyrate, propionate, and acetate metabolism, leading to VFA accumulation and the breakdown of microbial metabolic balance [2]. Ammonia inhibition also represents a major bottleneck, particularly for high-nitrogen wastes, because it selectively affects thermodynamically constrained and low-redundancy metabolic steps such as syntrophic propionate oxidation and acetoclastic methanogenesis [3]. Therefore, improving AD efficiency requires not only higher methane yield but also stronger resistance to metabolic imbalance and operational perturbations.

Among these stages, hydrolysis is widely recognized as the primary rate-limiting step, especially for sludge and lignocellulosic wastes. For instance, the dense recalcitrant structure of lignocellulosic matrices severely hinders hydrolytic enzyme penetration. Various pretreatment methods, including mechanical, thermal, chemical, and biological methods, have been extensively applied to enhance substrate solubilization and improve biogas production. Nevertheless, their practical application can be limited by energy input, chemical consumption, equipment cost, and the possible formation of inhibitory by-products. Beyond hydrolytic limitations, the accumulation of VFAs, ammonia, antibiotics, and other inhibitors can further suppress methanogenic activity and cause reactor acidification or collapse. These limitations highlight the need for strategies that can simultaneously improve substrate conversion, stabilize microbial metabolism, and enhance process resilience.

**Figure 1:**
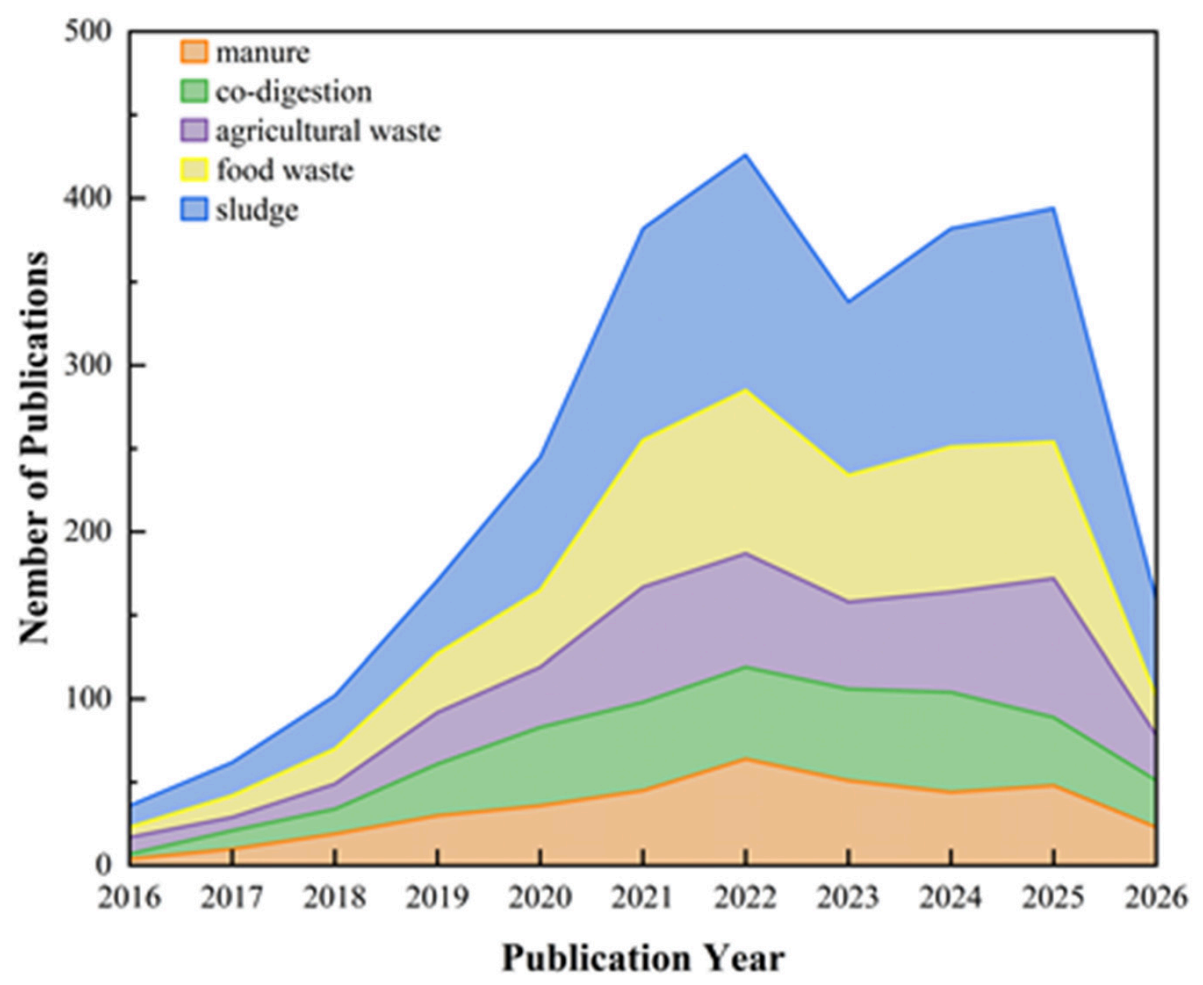
Number of publications associated with biochar-amended AD.

Currently, optimizing operational parameters and introducing functional additives (e.g., trace elements, adsorbents and carbon-based materials) represent the most viable strategy to enhance anaerobic digestion performance [4,5]. Among these additives, biochar has attracted attention because it is a stable, carbon-rich substance produced via the thermochemical conversion of waste biomass (e.g., pyrolysis, gasification, and hydrothermal carbonization) under oxygen-limited conditions. Compared with advanced carbon materials, such as graphene, carbon nanotubes and commercial activated carbon, biochar may not have the highest conductivity or specific surface area. Its practical advantage lies in the combination of relatively low cost, broad feedstock availability, and compatibility with circular waste management. This is primarily because it is directly derived from waste feedstocks. Its structural superiority stems from a large specific surface area, considerable porosity and abundant functional groups. Collectively, these features allow biochar to effectively adsorb inhibitory by-products and pollutants, thereby detoxifying fermentation. A large number of studies have confirmed that the addition of biochar significantly increased the cumulative methane production, shortened lag phases, improved VFA conversion and enriched syntrophic bacteria. These effects are often attributed to microbial immobilization, pH buffering, inhibitor adsorption, and direct interspecies electron transfer (DIET). For instance, Wang et al. [6] reported that the addition of biochar shortened the lag time by over 28% and increased biogas production by 22–40%. Magnetic biochar has been shown to enhance methane production and COD removal by combining the microbial attachment capacity of biochar with the electron-transfer activity of magnetite [7]. Similarly, magnetic biochar used in sludge AD produced synergistic effects on hydrolysis, acidogenesis, extracellular polymeric substance electroactivity, cytochrome c activity, ATP content, and the enrichment of electroactive microorganisms [8]. These findings indicate that biochar can regulate AD performance through multiple coupled pathways rather than through a single enhancement mechanism. Due to the above-mentioned superior characteristics and advantages, the addition of biochar in the AD system has received global attention, as presented in Figure 1.

Despite several studies summarizing biochar-assisted AD, most of them have focused mainly on methane enhancement, microbial community shifts, inhibition mitigation or DIET promotion. These studies have provided valuable knowledge, but important gaps remain. First, biochar properties are often discussed separately from AD mechanisms, making it difficult to explain why a certain biochar is effective under a specific operating condition [3,9,10,11,12,13,14]. The properties of biochar, such as porosity, conductivity, and surface functional groups, are highly tunable and strongly influenced by raw materials and synthesis parameters [15,16,17]. For instance, biochar produced at relatively low temperatures (approximately 300–500 °C) generally retains more functional groups, whereas it typically exhibits a higher fixed carbon content, large specific surface area and higher electrical conductivity at high temperature (approximately 600–900 °C). However, the key features related to enhancing AD performance remain unclear. Second, most evaluations focus on biogas yields and lag phases, neglecting the micro-dynamics of microbial succession, gene expression and electron transport pathways. In particular, DIET evidence is still frequently indirect, with many studies relying on microbial abundance, predicted functional genes and gas production rather than direct electrochemical or in situ interfacial measurements. Over 85% of studies claiming DIET enhancement base their conclusions solely on multi-omics data (e.g., a 30–50% increase in *Geobacter* abundance) and macroscopic gas metrics [18]. There is an extreme lack of direct in situ electrochemical evidence, such as conductive atomic force microscopy (c-AFM) or scanning electrochemical microscopy (SECM) [19]. Third, the possible trade-offs of biochar addition, including substrate adsorption versus nutrient deprivation, pore accessibility versus a large surface area, and redox modification versus chemical side effects, have not been sufficiently discussed. Finally, practical issues such as biochar aging, material recovery, long-term stability, digestate safety, life-cycle performance, and techno-economic feasibility remain underdeveloped in current discussions. Energy, CO_2_ emissions, and cost analyses further suggest that the integration of AD and biochar production should be evaluated from both environmental and economic perspectives before large-scale application.

Therefore, this review aims to provide a more integrated and critical understanding of biochar-assisted AD. Specifically, this study is structured around four objectives: (1) how feedstock selection, pyrolysis conditions, and modification strategies regulate the physicochemical properties of biochar; (2) evaluating the regulatory influence of biochar on AD performance, such as hydrolysis, VFA conversion, inhibition mitigation, etc.; (3) analyzing the underlying enhancement mechanism of biochar on the AD process; and (4) future research directions to overcome scale-up bottlenecks of biochar in AD systems. By linking biochar design with AD bottlenecks and practical implementation, this review seeks to clarify both the potential and the constraints of biochar as a functional material for resilient and resource-oriented anaerobic digestion.

## 2. Physicochemical Properties and Modulation of Biochar

Biochar has considerable potential as a functional additive in anaerobic digestion (AD), mainly because its physicochemical properties can be adjusted through feedstock selection and thermochemical conversion conditions. Pyrolysis process parameters, such as the type of feedstock, pyrolysis temperature, heating rate, residence time and post-modification, systematically affect the specific surface area, pore structure, surface functional groups, alkalinity and electrical conductivity of biochar. These properties are directly related to its roles in AD, including microbial immobilization, pH buffering, adsorption of inhibitory compounds, nutrient release, and interspecies electron transfer. Therefore, understanding how biochar properties are formed and how they regulate AD processes is essential for designing biochar with predictable and stable performance.

### 2.1. Feedstock Type and Composition

The type of raw materials used for biochar production significantly determines its elemental composition, surface chemistry and functional properties. Common biochar feedstocks include agricultural residues such as wood chips, animal manure, sewage sludge, food residues and digestate. Biochar derived from lignocellulosic biomass usually contains higher fixed carbon and develops stronger aromatic structures during pyrolysis. These characteristics make it more resistant to microbial degradation and suitable for long-term environmental applications, such as microbial attachment, electron transfer and carbon retention [9]. In contrast, biochar prepared from animal manure or sewage sludge usually has a higher ash content and inorganic mineral content, resulting in stronger alkalinity, higher buffering capacity, and greater potential for nutrient supply [20].

The intrinsic composition of the feedstock also affects the ion-exchange and adsorption properties of biochar. For example, hydrothermal biochar produced from cow manure has been reported to contain relatively low carbon but higher nitrogen than corn-straw-derived biochar, indicating that feedstock composition strongly affects the nutrient and surface chemical characteristics of the final material [21]. Biochar prepared from rice husks, coconut shells, and wood also shows different cation exchange capacities, suggesting that feedstock type can markedly influence the ability of biochar to retain ammonium, metal ions, and other charged species. Here, CEC refers to the total amount of exchangeable cations that can be retained on the biochar surface, rather than the exchange capacity of a specific cation. It has been reported that the cation exchange capacity (CEC) of biochar prepared from rice husks, coconut shells, and wood is 43.28 ± 1.49, 31.17 ± 0.35, and 21.47 ± 0.65 × 10^−2^ mmol/g, respectively. This indicated that the type of raw materials has a significant impact on the ionic exchange performance of biochar. Moreover, Oldfield et al. [22] prepared biochar using a mixture of plastic polymers and straw at 550 °C, significantly improving its physical and chemical properties, including specific surface area (16.4–27.5 m^2^/g), alkaline pH value (9.12–11.4), carbon content (55.8–58.1%), and a wide range of CEC values (43.28–321 × 10^−2^ mmol/g). Importantly, metal elements present in biomass (i.e., Ca^2+^, K^+^, Mg^2+^, and Fe^2+^) can further enhance the cation exchange capacity of biochar through surface complexation and electrostatic interactions, thereby improving its adsorption of nutrients, heavy metals, and inhibitory compounds.

For AD applications, the choice of feedstock should be matched with the target function. Lignocellulosic biochar is generally more suitable when microbial colonization, structural stability, or electron transfer is desired. Mineral-rich biochar from manure, sludge, or digestate may be more useful for buffering acidification and supplying trace elements. Recent studies have also emphasized the circular use of sludge- or digestate-derived carbon materials. Digestate-based biochar has been used to alleviate ammonia inhibition and improve microbial adaptation under high-ammonia conditions, while iron-oxide-modified digestate-derived biochar has been applied to stabilize high-loading kitchen waste digestion [23,24]. Hydrochar derived from wet sludge is another promising option because hydrothermal carbonization can directly process high-moisture feedstocks without energy-intensive drying and can retain abundant oxygen-containing functional groups that favor pollutant adsorption and microbial interaction [25]. However, low-temperature pyrolysis and hydrothermal carbonization products may contain residual soluble organic compounds, such as phenolic compounds, carboxylic acids, and other oxygen-containing molecules, which can be released during initial contact with aqueous systems. These leachable compounds may exhibit inhibitory effects on anaerobic microorganisms at high concentrations. Therefore, appropriate post-treatment procedures, such as water washing or aging, are recommended before biochar application in anaerobic digestion systems to remove potentially toxic soluble fractions and improve biocompatibility.

### 2.2. Pyrolysis Conditions

**Figure 2:**
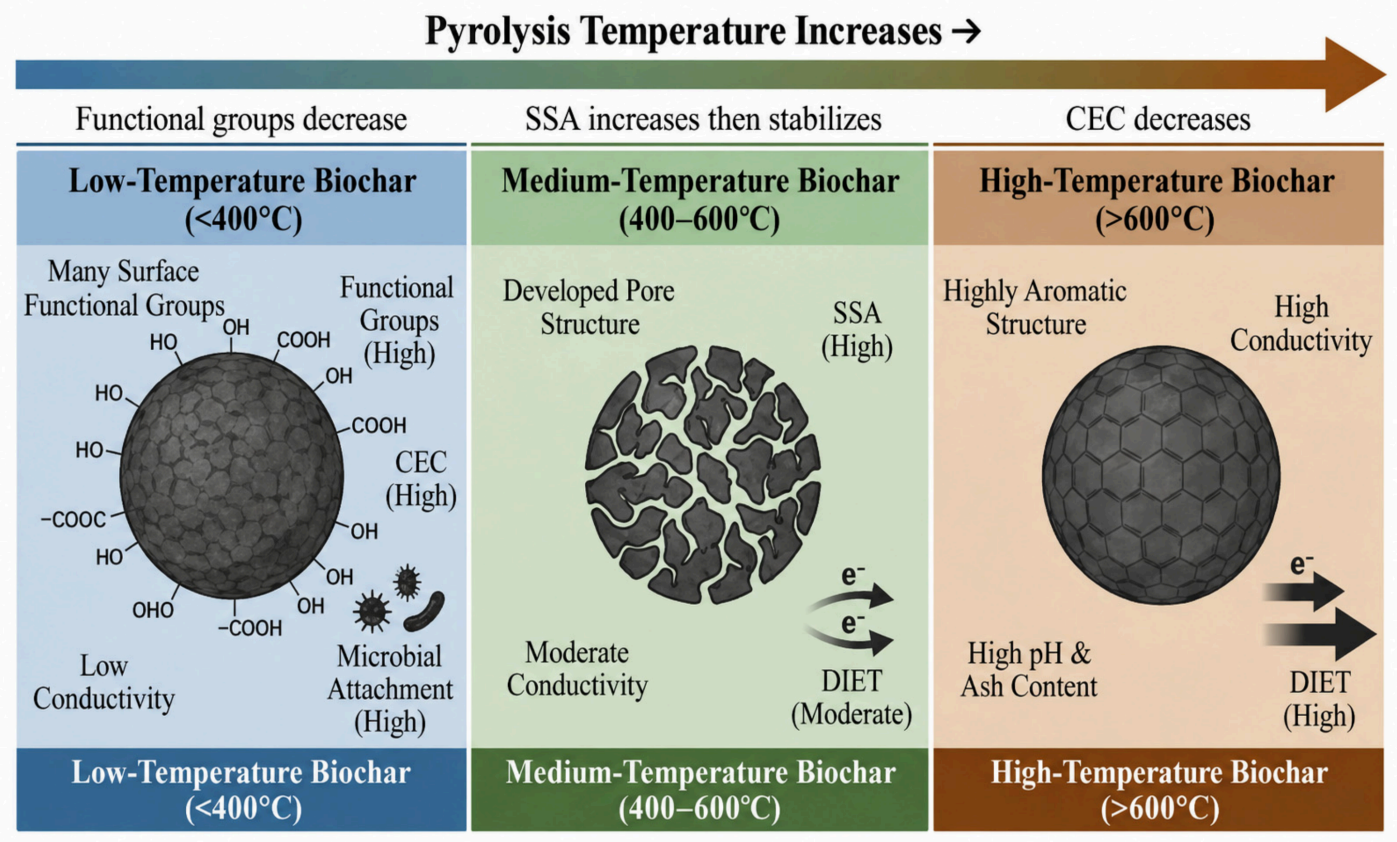
The relationship between the properties of biochar and pyrolysis temperature.

Pyrolysis temperature is one of the most important parameters that determine biochar properties, as shown in Figure 2. Pyrolysis at low-to-medium temperatures (300–500 °C) leads to the preservation of oxygen-containing functional groups, such as hydroxyl (-OH), carbonyl (-C=O-) and carboxyl groups (-COOH). These groups increase surface reactivity, cation exchange capacity and redox activity, which are beneficial for inhibitor adsorption, proton buffering and microbial interaction. In contrast, high temperatures above 600 °C promote dehydration, decarboxylation, aromatization, and graphitization. As a result, high-temperature biochar usually has higher fixed carbon content, larger aromatic domains, greater electrical conductivity, and a more developed pore structure, which may favor electron transfer and microbial colonization.

However, pyrolysis temperature should not be regarded as a simple “higher is better” parameter. High-temperature biochar may show improved conductivity and structural stability, but excessive carbonization can reduce polar functional groups and weaken ion exchange or redox-shuttling capacity. Conversely, low-temperature biochar may retain more functional groups, but it may also contain residual biodegradable components that complicate mechanistic interpretation in AD. Recent studies using negative controls have shown that part of the increase in methane observed with low-temperature biochar may arise from the biodegradable fraction of biochar itself rather than from direct interspecies electron transfer [26]. Therefore, the optimal pyrolysis temperature depends on the intended AD function, such as buffering, adsorption, microbial attachment, or electron transfer.

In addition to the pyrolysis temperature, the residence time and heating rate during pyrolysis also significantly affect the development of pore structure and material stability. A slower heating rate and sufficient residence time generally promote the formation of a stable pore system and a higher degree of carbonization. Different thermochemical routes may produce biochar with substantially different properties. For example, Mumme et al. [27] reported that pyrolyzed biochar prepared at 500 °C had a relatively high ash content of 39.2% *w*/*w*, a dry matter content of 54.7% *w*/*w*, and was alkaline at a pH of 9.3. In contrast, hydrochar produced by hydrothermal carbonization at 230 °C had a lower ash content of 7.8% *w*/*w*, a dry matter content as high as 99.8% *w*/*w*, and a pH of 4.8, showing obvious acidic characteristics. This distinction is important because pyrolyzed biochar and hydrochar may behave differently in AD, which is consistent with the findings of Ren et al. [28] and Yin et al. [29].

The performance of biochar in anaerobic digestion is strongly dependent on both feedstock characteristics and pyrolysis conditions. Biochars derived from lignocellulosic biomass, such as crop residues (rice husk, corn straw) and wood, generally possess stable carbon structures and developed pore networks, making them suitable for microbial immobilization and electron transfer enhancement. In contrast, manure-, sewage sludge-, and digestate-derived biochars contain abundant mineral components (e.g., Ca, Mg, Fe, and P), which can provide buffering capacity and nutrient supplementation under anaerobic digestion conditions. Polymer-containing biochars and activated biochars usually exhibit enhanced surface area and conductivity, but their application requires careful evaluation due to possible residual contaminants.

Pyrolysis temperature is another critical factor determining biochar functionality. Low-temperature pyrolysis (typically <500 °C) preserves abundant oxygen-containing functional groups, improving hydrophilicity and microbial interaction; however, it may result in the release of soluble organic compounds, including phenolic compounds and organic acids, which could inhibit anaerobic microorganisms if not removed. High-temperature pyrolysis (>600 °C) promotes aromatic carbon formation, higher conductivity, and improved electron transfer capacity, but excessive carbonization may decrease surface functional groups and reduce biological affinity. Therefore, the optimal biochar preparation conditions should be selected according to the intended application.

### 2.3. Key Physicochemical Characteristics Relevant to AD

(1) Specific surface area and porosity

The specific surface area and pore structure of biochar are important for microbial attachment, biofilm formation, and microbe contact with substrate. The porous structure of the biochar can provide anchoring sites for hydrolytic bacteria, syntrophic bacteria and methanogenic archaea, thereby improving biomass retention and protecting functional microorganisms from washout and toxic shocks [30].

Biochar pore systems generally consist of micropores (<2 nm), mesopores (2–50 nm), and macropores (>50 nm), which play different roles in anaerobic digestion applications. Micropores mainly contribute to large specific surface areas and adsorption of small molecules, whereas mesopores facilitate substrate diffusion and adsorption of larger compounds. Macropores provide transport channels and larger spaces for microbial attachment and biofilm development. The distribution and connectivity of these pores vary considerably among biochars depending on feedstock composition, pyrolysis temperature, and activation methods. For example, high-temperature pyrolysis often increases aromaticity and micropore formation, while excessive carbonization may reduce oxygen-containing functional groups and microbial affinity. Therefore, pore size distribution and accessibility under hydrated anaerobic conditions should be considered together with BET surface area when evaluating biochar performance in AD systems. In addition to pore structure, particle size distribution is another important factor determining the effectiveness of biochar in AD systems. Smaller biochar particles generally exhibit a larger external surface area and shorter diffusion distances, which can enhance microbial attachment, substrate interaction, and electron transfer. However, excessively small particles may promote aggregation, block pores, reduce mass transfer efficiency, and increase operational difficulties during reactor mixing or separation. Larger biochar particles provide more stable physical structures for biofilm development and may improve long-term stability, but their lower accessible surface area may limit adsorption and microbial contact. Therefore, the optimal particle size of biochar should be selected according to the targeted function, reactor configuration, and microbial requirements.

Nevertheless, a specific surface area alone is not sufficient to predict AD enhancement. Very small micropores may contribute to gas or small-molecule adsorption but may not be accessible to microbial cells or extracellular enzymes. In this case, the apparent surface area may be large, while the biologically effective surface area is limited. Recent work on tubular-pore biochar further showed that pore architecture, pore uniformity, and microbial accessibility can be more important than total specific surface area in regulating microbial enrichment, hydrolysis, and electron transfer [10,30]. Therefore, future biochar characterization for AD should not only include BET surface area, but also include pore size distribution, pore connectivity, and accessibility under wet anaerobic conditions.

Besides serving as a microbial carrier, biochar can act as an efficient adsorbent for harmful gas components in biogas [31]. Laboratory-scale studies have shown that four types of biochar were evaluated for their removal effects on CO_2_ and H_2_S, achieving 0.208 mmol/g and 0.126 mmol/g respectively. Similarly, Creamer et al. [32] found that biochar prepared from pecan wood and sugarcane residues can achieve effective CO_2_ adsorption driven by a large specific surface area and nitrogen functional groups. Pore size ranges of approximately 0.5–0.8 nm [33] and large specific surface sites [34] are considered important for CO_2_ sequestration, while the presence of alkali metals and surface basicity can further enhance adsorption performance [35]. These properties suggest that biochar may contribute not only to AD performance but also to in situ biogas upgrading.

(2) Electrical conductivity

Electrical conductivity is frequently discussed as a key property of biochar because conductive biochar can facilitate direct interspecies electron transfer (DIET) between syntrophic bacteria and methanogens. High-temperature pyrolysis, graphitization, and heteroatom doping can increase conductivity and reduce charge-transfer resistance. Conductive biochar may therefore act as an electron pathway that connects electroactive bacteria with methanogens, accelerating the conversion of VFAs to methane. However, the electrical conductivity (EC) of biochar itself usually does not dominate AD performance, but its contribution depends on substrate composition, microbial community and the presence of redox-active surface moieties [36,37]. Even biochar with relatively low conductivity can promote methanogenesis if it provides suitable microbial attachment sites or redox-active functional groups [35]. Recent studies also suggest that electron transfer may occur through electron hopping mediated by cytochrome c and riboflavin-like redox units at the biochar–microbe interface, rather than through bulk conductivity alone [38]. Therefore, conductivity, electron exchange capacity, and surface redox chemistry should be evaluated together when discussing biochar-mediated electron transfer.

(3) pH and buffering capacity

Biochar is usually alkaline because pyrolysis enriches ash minerals and removes acidic functional groups. With increasing pyrolysis temperature, biochar pH often increases because of the accumulation of carbonates, oxides, and alkaline mineral species [28,29]. In AD, alkaline biochar can buffer acidification by neutralizing protons, increasing alkalinity, and supporting bicarbonate reserves. This is particularly important under high organic loading or rapid VFA accumulation, where the decline in pH can inhibit methanogens and cause process failure. Previous studies have shown that adding biochar can significantly increase alkalinity, thereby accelerating the CH_4_ generation rate and improving the reactor’s adaptability under organic load shock [39]. For example, walnut shell biochar increases the system’s alkalinity from 2800 mg/L to 4800 and 6800 mg/L under mesophilic and thermophilic conditions [40]. Sewage sludge biochar has also been reported to accelerate VFA degradation and methane generation [41]. In addition to mineral alkalinity, surface functional groups such as hydroxyl, carboxyl, and phenolic groups can participate in proton exchange and reversible redox reactions, contributing to pH buffering and electron-shuttling capacity.

(4) Surface functional groups

The biochar surface contains various oxygen-containing functional groups, including hydroxyl groups (-OH), carbonyl groups (-C=O), carboxyl groups (-COOH), and phenolic and quinone-like groups. These functional groups provide active sites for adsorption, ion exchange, complexation, hydrogen bonding and redox reactions. In AD, they can reduce the bioavailability of inhibitory compounds, such as NH_4_^+^, H_2_S, antibiotics and heavy metals. For example, NH_4_^+^ can bind to acidic functional groups through the ion exchange mechanism, while higher cation exchange capacity can improve ammonium retention and alleviate ammonia inhibition [42,43,44]. In addition, H_2_S removal can be promoted by surface functional groups (i.e., -OH and -COOH), which provide active sites for chemical capture and catalytic oxidation [45,46]. Recent work suggests that different functional groups may regulate different AD steps. Hydroxyl-rich biochar has been linked to improved methanogenic electron transfer, whereas carboxyl-rich biochar may favor organic matter decomposition [47]. This indicates that surface functional groups should not be treated as a general descriptor, but rather as targeted properties that may influence hydrolysis, acidogenesis, inhibition mitigation, or methanogenesis in different ways.

### 2.4. Biochar Modification and Functionalization

The physical and chemical properties of biochar are largely determined by the composition of the raw materials and the pyrolysis conditions (Table 1). It is difficult to predict the effect of unmodified biochar in the AD system. Their effects depend on the target bottleneck of the digester, such as hydrolysis limitation, acidification, ammonia inhibition, VFA accumulation, or inefficient syntrophic electron transfer. Modification and functionalization strategies have therefore been developed to regulate specific properties, including pore structure, surface functional groups, conductivity, redox activity, and mineral composition. Common methods include physical activation, chemical modification, heteroatom doping, and mineral loading.

Physical activation and high-temperature pyrolysis primarily drive the textural and structural properties of biochar. These methods increase specific surface area and promote graphitization, thereby creating a porous and conductive scaffold for microbial attachment, biofilm formation, and DIET [48]. Chemical modification mainly focuses on regulating surface chemistry. By adjusting the speciation of oxygen-containing functional groups, these treatments optimize the redox activity and cation exchange capacity of the material, thereby enhancing the adsorption and buffering effects on inhibitory substances. However, chemical modification should be used cautiously. Inappropriate functionalization may introduce residual chemicals, cause pH disturbance, release metals, or generate inhibitory substances. For example, quinone-functionalized algal biochar enhanced acidogenesis but inhibited methane production, probably because the modification process introduced unfavorable chemical side effects [49].

Mineral-modified biochar, with Fe, Mn or alkaline earth metals, further enhances the redox and buffering function of biochar. Iron-loaded and magnetic biochar can promote electron transfer, enrich electroactive microorganisms, and facilitate material recovery. Magnetic biochar has been reported to generate synergistic effects between the carbon matrix and magnetite, improving hydrolysis, acidogenesis, extracellular polymeric substance electroactivity, cytochrome c activity, ATP supply, and methanogenic performance [8]. Heteroatom-doped biochar also provides a route to tune electrochemical properties. For instance, phosphorus-doped iron-loaded biochar showed enhanced methane production and enriched Methanobacterium, suggesting that capacitance and electron-transfer resistance can be regulated through targeted material design [7].

**Table 1:** Linkages between the biochar properties, regulation strategies, AD mechanisms and functional outcomes.

Biochar Property	How the Property Is Regulated	Functional Outcome in AD	Main AD-Related Mechanisms	Limitation	Reference
Specific surface area (SSA) and accessible porosity	Physical activation, controlled pyrolysis temperature; pore-forming feedstocks	Improves biomass retention, hydrolysis efficiency, and syntrophic contact	Provides more attachment sites for syntrophic microbes and methanogens	High BET surface area alone does not guarantee AD enhancement; very small micropores may be inaccessible to microorganisms	Refs. [30,32,50]
Porous structure	Selection of tubular or fibrous biomass; particle-size control	Improves methane production stability	Creates microbial microhabitats and improves local retention of substrates, enzymes and intermediates	Pore size distribution and biological accessibility may be more important than total surface area	Refs. [50,51]
Electrical conductivity	High-temperature pyrolysis, graphitization, N/P/S doping and carbon–metal composite formation	Enhances VFA conversion and methane production rate	Improves direct interspecies electron transfer (DIET) between syntrophic partners	Bulk conductivity is not the only predictor; redox-active groups and microbial contact also matter	Refs. [38,43]
Surface functional groups	Low-/medium-temperature pyrolysis, oxidation, acid/alkali treatment	Enhances adsorption of NH_4_^+^ and H_2_S and antibiotics and metals; reduces inhibition	Provides active sites for ion exchange, hydrogen bonding, complexation and redox mediation	Different groups may affect different AD stages; functional groups should not be treated as a single general descriptor	Ref. [46]
Cation exchange capacity (CEC)	Feedstock mineral composition, surface oxidation, chemical activation	Retains NH_4_^+^ and other charged species through ion exchange and electrostatic interaction	Alleviates ammonia inhibition and improves buffering capacity	Excessive adsorption may reduce the availability of essential nutrients	Refs. [23,44]
Redox-active moieties (quinone/phenolic groups)	Preservation or introduction of quinone, phenolic, carbonyl, and other oxygen-containing groups	Accelerates syntrophic metabolism and stabilizes electron flow	Acts as an electron shuttle or transient electron sink at the biochar and microbe interface	Excessive or harsh functionalization may introduce inhibitory effects	Refs. [38,49,52]
Mineral ash and alkalinity	Manure-, sludge-, or digestate-derived feedstocks; higher pyrolysis temperature; alkaline mineral loading	Maintains favorable pH and improves resistance to acidification and organic loading shocks	Releases alkaline minerals and replenishes buffering capacity	Excessive ash or metal release may affect microbial activity and digestate safety	Refs. [20,23,53]
Fe/Mn-containing phases and magnetic components	Inherent Fe-rich feedstocks, Fe/Mn loading, magnetization, ball milling	Enhances methanogenesis, VFA degradation, and material separation	Promotes redox cycling, electron transfer, and possible DIET; enables magnetic recovery	High metal loading may block pores or cause metal leaching	Refs. [7,8,54]
Particle size and spatial distribution	Grinding, pelletization, granulation, mixing intensity, reactor configuration	Influences reactor stability and biogas production under semi-continuous operation	Affects dispersion, microbial colonization, mass transfer, and contact between biochar and biomass	Smaller particles improve dispersion but may be difficult to recover and may intensify microbial over-colonization	Ref. [51]
Feedstock-derived nutrients and circular carbon source	Use of sludge-, manure-, or digestate-derived biochar/hydrochar	Supports waste valorization, ammonia-stress mitigation, and internal recycling of AD by-products	Supplies nutrients, trace elements, alkalinity, and microbial attachment sites	Potential risks include heavy metals, PAHs, and digestate safety concerns	Refs. [23,24,25]

Overall, biochar modification should be guided by the target bottleneck of the AD system. For acidification, mineral-rich or alkaline biochar may be preferred. For ammonia or sulfide inhibition, biochar with high cation exchange capacity and suitable surface functional groups is more relevant. For slow syntrophic metabolism, conductive or redox-active biochar may be more effective. For engineering applications, magnetic, granular, or immobilized biochar may be more practical because of improved recovery and retention. Thus, the key challenge is not simply to produce stronger biochar, but to match biochar properties with specific AD requirements [48].

## 3. Performance Enhancement and Mechanisms of Anaerobic Digestion by Biochar

**Figure 3:**
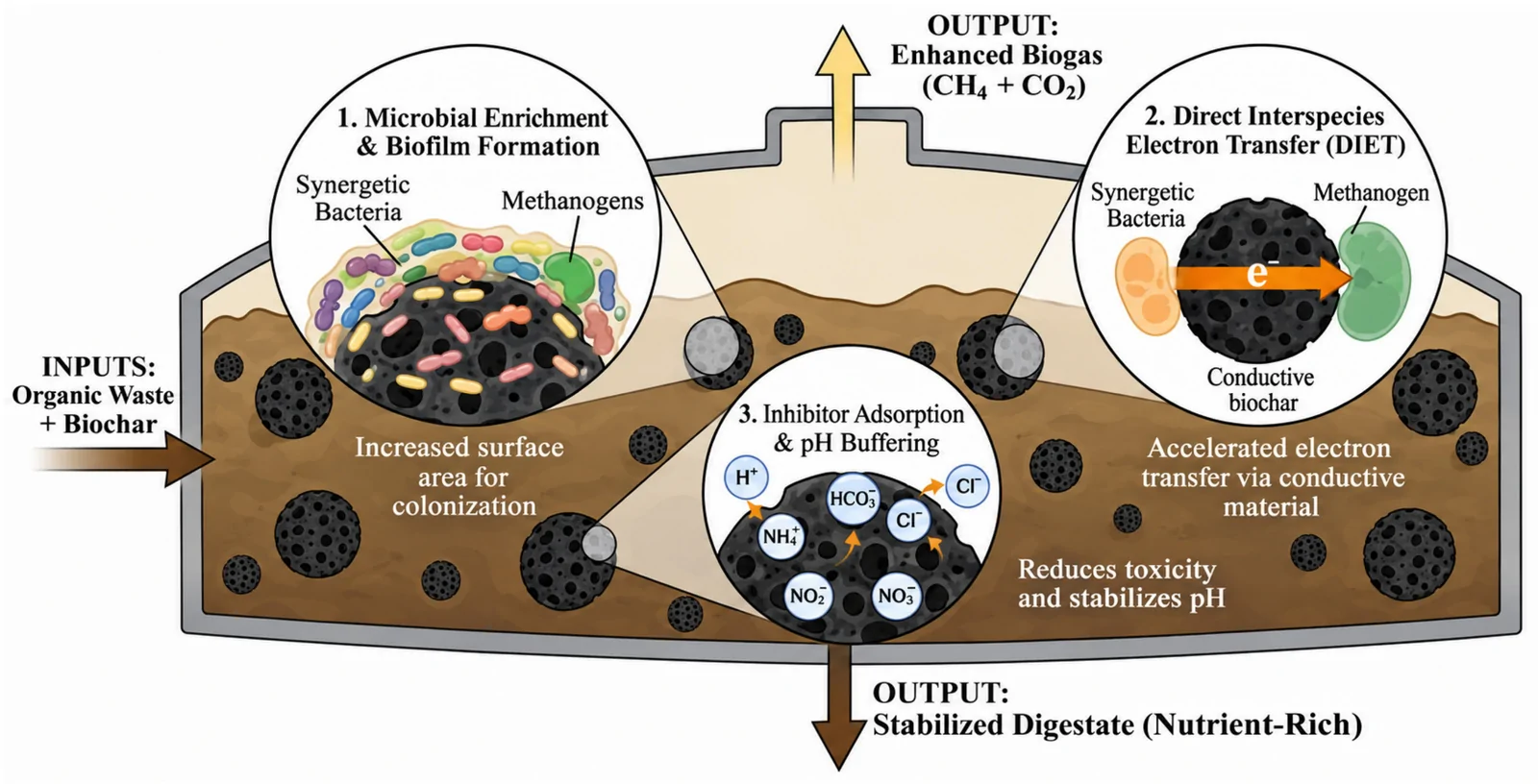
Main pathway through which biochar is enhanced in the AD process.

Biochar intervention has emerged as a pivotal strategy to enhance AD performance, but its effect should not be interpreted as a single methane enhancement mechanism. Instead, biochar regulates AD through several pathways, including physicochemical buffering, inhibitor adsorption, microbial immobilization, syntrophic metabolism, and interspecies electron transfer. These functions are closely related to the properties discussed above, such as pore structure, alkalinity, surface functional groups, mineral composition, conductivity, and redox-active moieties. This section discusses the role of biochar in methane production, process stabilization, inhibition mitigation, microbial community regulation and DIET, as shown in Figure 3.

### 3.1. Methane Production and Energy Recovery

Methane production is the most frequently used indicator for evaluating biochar-assisted AD. Existing studies have shown that the addition of biochar can enhance methane recovery from a wide range of substrates, including sewage sludge, livestock manure, lignocellulosic residues, food waste, and algal biomass. For example, methane yield improvements of 8.6% to 17.8% have been reported during sewage sludge digestion [53]. Higher enhancement has been observed for high-strength substrates, such as the chicken manure digestion system, which surged by 32–36% [21] and food waste and algal biomass, which achieved 46.9% [55] and 35–37% [56], respectively. Specifically, biochar with its large specific surface area and well-developed porous structure provides a large number of attachment sites for hydrolytic bacteria and extracellular hydrolases. However, these reports should be interpreted together with the corresponding substrate characteristics, biochar properties, and reactor conditions, rather than being treated as directly comparable values.

The enhancement of methane production by biochar is first related to improvements in substrate conversion. The hydrolysis process acts as the primary kinetic bottleneck in the AD process. This rate-limiting step is particularly severe for complex organic substances, such as lignocellulosic biomass and EPS-dense sludge. Biochar with a rough surface and developed pore structure can provide attachment sites for hydrolytic bacteria and extracellular enzymes, increasing the local contact between enzymes and particulate substrates. In addition, functional groups on the surface of biochar (e.g., hydroxyl groups, carboxyl groups and aromatic structures) help to stabilize the adsorbed hydrolytic enzymes and prolong their catalytic activity. In this way, biochar can promote the solubilization of proteins, polysaccharides, and lipids, thereby increasing the availability of substrates for acidogenesis and methanogenesis [57].

Biochar may also enhance methane production by improving the balance between acidogenesis and methanogenesis. During AD of easily degradable wastes such as food waste, rapid acidogenesis can lead to VFA accumulation and pH decline. Biochar can buffer this imbalance by adsorbing VFAs, releasing alkaline minerals, supporting methanogenic colonization, and facilitating electron transfer between syntrophic bacteria and methanogens. Recent studies further suggest that biochar-mediated methane enhancement may depend on more specific material features. For example, tubular-pore biochar was shown to improve methane production not simply because of its larger specific surface area, but because of its pore structure, microbial accessibility, and enhanced hydrolysis–methanogenesis coupling [50]. Similarly, magnetic biochar may enhance methane recovery through the combined effects of microbial attachment, redox cycling, and electroactive microbial enrichment [7,8].

Nevertheless, biochar does not always increase methane production under all conditions. Excessive addition may lead to pore blockage or hinder material transfer, reducing the effective contact area between microorganisms and substrates. In some cases, methane enhancement may also be partly caused by the residual biodegradable fraction of low-temperature biochar rather than DIET or conductivity-driven mechanisms. However, over-adsorption of soluble organics by biochar can inadvertently starve methanogens, leading to inhibited methane production [58]. Therefore, methane production should be discussed as an integrated outcome of substrate characteristics, biochar properties, microbial activity, and reactor operation.

Overall, biochar can improve methane recovery by enhancing hydrolysis, stabilizing VFA conversion, supporting methanogens, and facilitating syntrophic metabolism. However, the reported enhancement effects vary greatly in different studies due to the fact that biochar efficacy is highly dependent on the specific coupling of material characteristics, substrate composition, and reactor hydrodynamic conditions. Therefore, it is necessary to systematically clarify the interrelationships among these key factors to promote the reliable application and large-scale promotion of biochar in anaerobic digestion.

### 3.2. Process Stabilization of System and Lag Period Shortening

Process stability is a critical factor for AD operation. The AD system is susceptible to a variety of operating parameters, including temperature fluctuation, organic loading rate (OLR), VFA accumulation, and hydrogen partial pressure. These disturbances can disrupt the balance between acid-producing microorganisms and methanogens, leading to acidification, delayed methane production, and even reactor failure. The introduction of biochar can enable the system to maintain stable operation by buffering pH, adsorbing inhibitory intermediates, providing microbial habitats, and supporting syntrophic electron transfer.

Temperature fluctuation is one factor that can affect microbial kinetics and methane production. Although most digesters are operated under mesophilic or thermophilic conditions, unexpected temperature variation may reduce methanogenic activity. Previous studies have shown that biochar can help maintain VFA conversion and methane production under suboptimal or thermophilic conditions, possibly through microbial retention, buffering capacity, and enhanced syntrophic metabolism [59,60]. However, the stabilizing effect depends on the substrate, biochar type, and microbial adaptation, and therefore should not be generalized without considering reactor conditions.

High organic loading (OLR) operations are attractive because they can reduce reactor volume and improve volumetric productivity, but organic overload frequently triggers rapid VFA accumulation and a decrease in pH. Biochar can alleviate these effects by adsorbing VFAs, buffering acidity, and improving the conversion of propionate and butyrate. Under overload conditions, the degradation of VFAs is thermodynamically constrained and depends strongly on syntrophic interactions. By supporting close contact between syntrophic bacteria and methanogens, biochar can reduce the accumulation of intermediates and maintain more favorable conditions for VFA oxidation [61,62]. Recent metagenomic evidence also indicates that overload-induced instability is closely associated with the blockage of butyrate, propionate, and acetate metabolic pathways, further emphasizing the importance of stabilizing VFA conversion [2,63].

Biochar can also shorten the lag phase during AD start-up. The lag phase is often prolonged by substrate toxicity, insufficient methanogenic biomass, or delayed microbial adaptation. Biochar provides microbial attachment sites and protects cells from environmental shocks, which can accelerate the establishment of active syntrophic consortia. Even under severe dual inhibition (i.e., ammonia and VFA), biochar has been proven to decrease the lag period by nearly 24% [64]. This effect is particularly pronounced in complex co-digestion conditions (e.g., sludge and food waste), where latency reductions of 27.5–64.4% have been recorded [65]. Similar effects have also been observed in systems treating lipid-rich waste [58], lignocellulosic residues [66], beer mash [67], and toxic industrial streams like phenolic wastewater [68] and landfill leachate [69]. These results indicate that biochar can facilitate the transition from microbial acclimation to active methane production.

In addition, biochar also functions as a pH buffer, preventing catastrophic acidification in unstable AD systems. Unlike instantaneous chemical dosing, biochar provides sustained pH regulation through alkaline minerals and surface functional groups [70]. Minerals such as Ca^2+^, Mg^2+^, and K^+^ can replenish alkalinity, while surface groups such as -OH and amino (-NH_2_) groups can participate in proton exchange [71,72]. Concurrently, biochar indirectly reduces the solution’s acidity by physically adsorbing free VFAs [73].

In summary, process stability, by adding biochar, is not only attributed to increasing methane yield, but also to enhancing resistance to acidification, organic overload, VFA accumulation and microbial imbalance. However, long-term evidence from continuous reactors is still limited, and future work should determine how biochar aging, particle retention, and reactor hydrodynamics affect its stabilizing function during extended operation.

### 3.3. Mitigation of Inhibition by Toxic Compounds

The accumulation of inhibitory compounds is considered to be a primary cause of AD destabilization. Common inhibitors include ammonia, sulfide, VFAs, heavy metals, phenolic compounds, antibiotics, and other recalcitrant organic pollutants. These inhibitors exert their toxicity by disrupting cellular membranes or binding to active enzymatic sites [74,75]. Biochar can mitigate inhibition, mainly through adsorption, ion exchange, surface complexation, precipitation, buffering, and microbial protection.

The adsorption capacity of biochar is closely related to its pore structure, surface functional groups, mineral composition, and cation exchange capacity. The multi-level pore structure formed during pyrolysis provides a large number of micropores and mesopores, which are conducive to the multilayer adsorption and pore filling of soluble inhibitors. Meanwhile, the oxygen-rich functional groups (e.g., hydroxyl, carboxyl, carbonyl, and phenolic hydroxyl groups) on the surface of biochar can participate in the fixation of pollutants through various mechanisms, including electrostatic interaction, hydrogen bonding, ligand coordination, π-π interaction, and hydrophobic partitioning. Through these mechanisms, biochar can reduce the free concentration and bioavailability of inhibitors in the liquid phase.

For inorganic species, NH_4_^+^ can be retained through cation exchange with minerals (e.g., Ca^2+^, Mg^+^, and K^+^) on the surface of biochar [76]. This process can reduce ammonia stress and improve the tolerance of methanogens. Heavy metals can be immobilized through surface complexation, carbonate precipitation, and adsorption onto mineral phases [77]. H_2_S and HS^−^ can also be captured by alkaline minerals and surface functional groups, reducing sulfide toxicity and improving biogas quality [78]. However, the adsorption of inhibitors should be balanced with the retention of nutrients because excessive or non-selective adsorption may reduce the availability of trace elements needed for methanogenic enzymes.

Biochar can also reduce inhibition caused by organic pollutants and antibiotics. Phenolic compounds, furfural, chlorophenols and antibiotics may inhibit syntrophic bacteria and methanogens, leading to VFA accumulation and reduced methane production [79]. Functionalized biochar can lower the bioavailability of these compounds through adsorption. In some cases, it promotes their transformation through redox-active or metal-containing sites. For instance, Fe_3_O_4_-modified biochar has been shown to simultaneously depurate aniline and enhance methanogenesis in dye wastewater [80]. More recent studies showed that iron-rich or zero-valent-iron-modified biochar can mitigate antibiotic stress by combining adsorption, pH buffering, and electron-transfer recovery [19,81]. In swine wastewater AD under ciprofloxacin stress, cow-dung-derived biochar had inherent iron-enhanced methane production, enriched Clostridium and *Methanothrix*, and improved acidogenesis and methanogenesis [82]. In aquaculture wastewater containing oxytetracycline and sulfamethoxazole, zero-valent iron-modified biochar reduced antibiotic bioavailability and helped maintain acetoclastic methanogenesis under combined antibiotic stress [83].

Despite these advantages, engineered biochar should be used cautiously. Chemical modification may improve adsorption or redox activity, but it may also introduce residual reagents, pH disturbance, metal leaching, or inhibitory substances. For example, quinone-functionalized biochar improved acidogenesis but inhibited methane production in one study, indicating that redox-active modification does not necessarily lead to better AD performance [49]. Therefore, future studies should evaluate not only inhibitor removal efficiency but also microbial activity, methane production, nutrient availability, and potential secondary risks.

In summary, biochar can serve as a broad-spectrum resilience shield against chemical stressors in AD. Its inhibition-mitigation effect is strongest when surface chemistry, mineral composition, and pore structure are matched with the dominant inhibitor. Therefore, future research should focus on balancing toxin sequestration with nutrient preservation and avoiding introducing new risks during biochar modification.

### 3.4. Regulation of Microbial Community and Metabolic Pathways

Biochar not only serves as a passive immobilization carrier, but it actively constructs a favorable micro-niche for colonization and syntrophic interaction. Compared with traditional immobilization methods (e.g., gel entrapment or inert carrier adsorption), biochar has a rough surface, porous structure, functional groups, mineral nutrients, and redox-active sites. These properties promote the spontaneous formation of dense, active biofilms, protect biomass from washout and improve microbial tolerance to pH shocks and toxic inhibitors [84]. As summarized in Table 2, biochar-induced microbial regulation is not limited to the enrichment of electroactive microorganisms. Depending on the substrate and biochar design, biochar can promote hydrolytic and fermentative bacteria, VFA-degrading syntrophs, acetoclastic and hydrogenotrophic methanogens, and stress-adaptive microbial functions. Therefore, microbial responses should be interpreted together with reactor conditions and material properties rather than being used alone as direct evidence for DIET.

**Figure 4:**
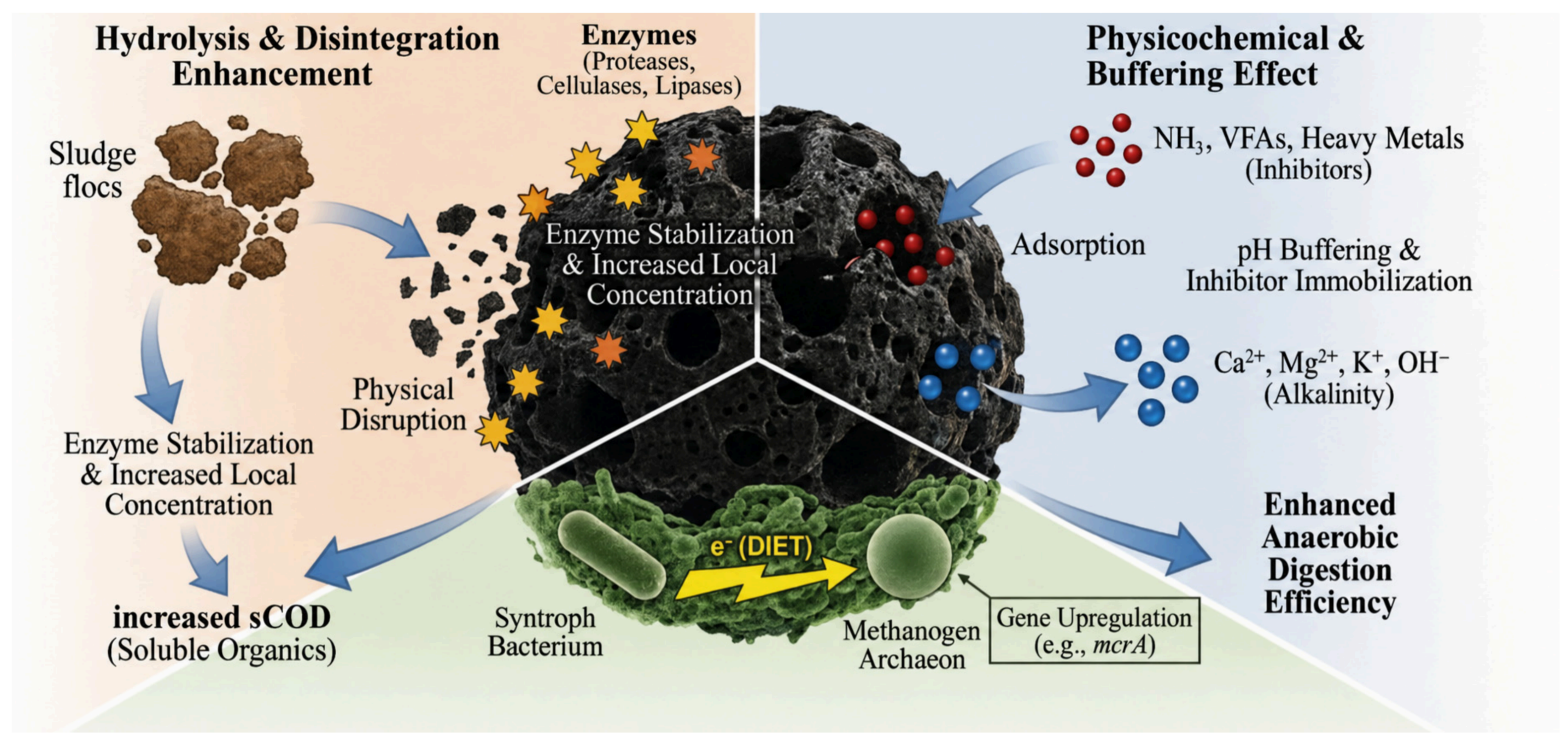
Microbial regulation mechanisms of biochar on the anaerobic digestion process.

Structurally, biochar enforces a spatial confinement effect that reshapes syntrophic interactions, as shown in Figure 4. The porous structure of biochar provides spatial niches for hydrolytic bacteria, fermentative bacteria, syntrophic acetogens, and methanogenic archaea. By co-localizing these functional groups within or near its pore network, biochar can shorten the diffusion distance of key intermediates such as H_2_, formate, acetate, and VFAs. This spatial proximity favors syntrophic metabolism and helps maintain local hydrogen partial pressure at thermodynamically favorable levels [85]. Furthermore, the in situ leaching of trace elements (e.g., Fe, Co, and Ni) from biochar creates localized micronutrient hotspots for hydrogenases, dehydrogenases, and methanogenic enzymes, thereby supporting metabolic activity under stress conditions [86,87].

The effects of biochar on microbial communities are species-dependent. Conductive biochar can selectively enrich electroactive microorganisms, such as Geobacter, and syntrophic bacteria by facilitating extracellular electron transfer. Meanwhile, DIET-associated methanogens, including *Methanothrix* and *Methanosarcina*, may benefit from enhanced electron exchange pathways. However, not all microorganisms respond positively to biochar addition. Soluble organic compounds released from low-temperature biochar, such as phenolic compounds and organic acids, may inhibit sensitive methanogenic populations during the initial adaptation stage. Therefore, biochar selection should consider both the desired microbial functions and potential inhibitory effects.

Biochar can also reshape the microbial community structure. Studies have shown that biochar addition can enrich hydrolytic bacteria, VFA-degrading bacteria, electroactive bacteria, and methanogenic archaea. For example, Clostridium, Syntrophomonas, Geobacter, Methanosaeta, *Methanosarcina*, and Methanobacterium are frequently reported in biochar-amended systems, depending on substrate type and operating conditions [84,85,86]. Biochar can also affect the abundance of genes related to hydrolysis, acidogenesis, methanogenesis, and extracellular electron transfer. However, these microbial changes should be interpreted carefully. The enrichment of electroactive microorganisms or genes such as pilA, omcS, and mcrA may suggest enhanced electron transfer [67,90], but it does not by itself prove DIET. Direct electrochemical evidence and interfacial measurements are still needed.

Recent studies have expanded the microbial interpretation of biochar-assisted AD. In high-loading or collapsed digesters, magnetic biochar combined with digestate bioaugmentation enriched DIET-associated microorganisms, including *Methanosarcina* and Geobacter, and accelerated reactor recovery [91]. In propionate-stressed systems, a mycelial pellet–biochar composite carrier enhanced methane production by supporting microbial immobilization, osmoregulation, quorum sensing, protein synthesis, repair processes, and ATP synthesis [92]. These findings suggest that biochar may regulate not only community composition but also microbial physiological activity and stress adaptation.

Therefore, biochar-mediated microbial regulation should be understood as a combination of physical retention, niche construction, nutrient supply, toxicity mitigation, and metabolic stimulation. Future studies should avoid relying solely on community abundance changes and should combine metagenomics, metatranscriptomics, metaproteomics, enzyme assays, electrochemical measurements, and isotope tracing to identify the real contribution of each pathway.

### 3.5. Direct Interspecies Electron Transfer (DIET) and Electrochemical Mediation

**Figure 5:**
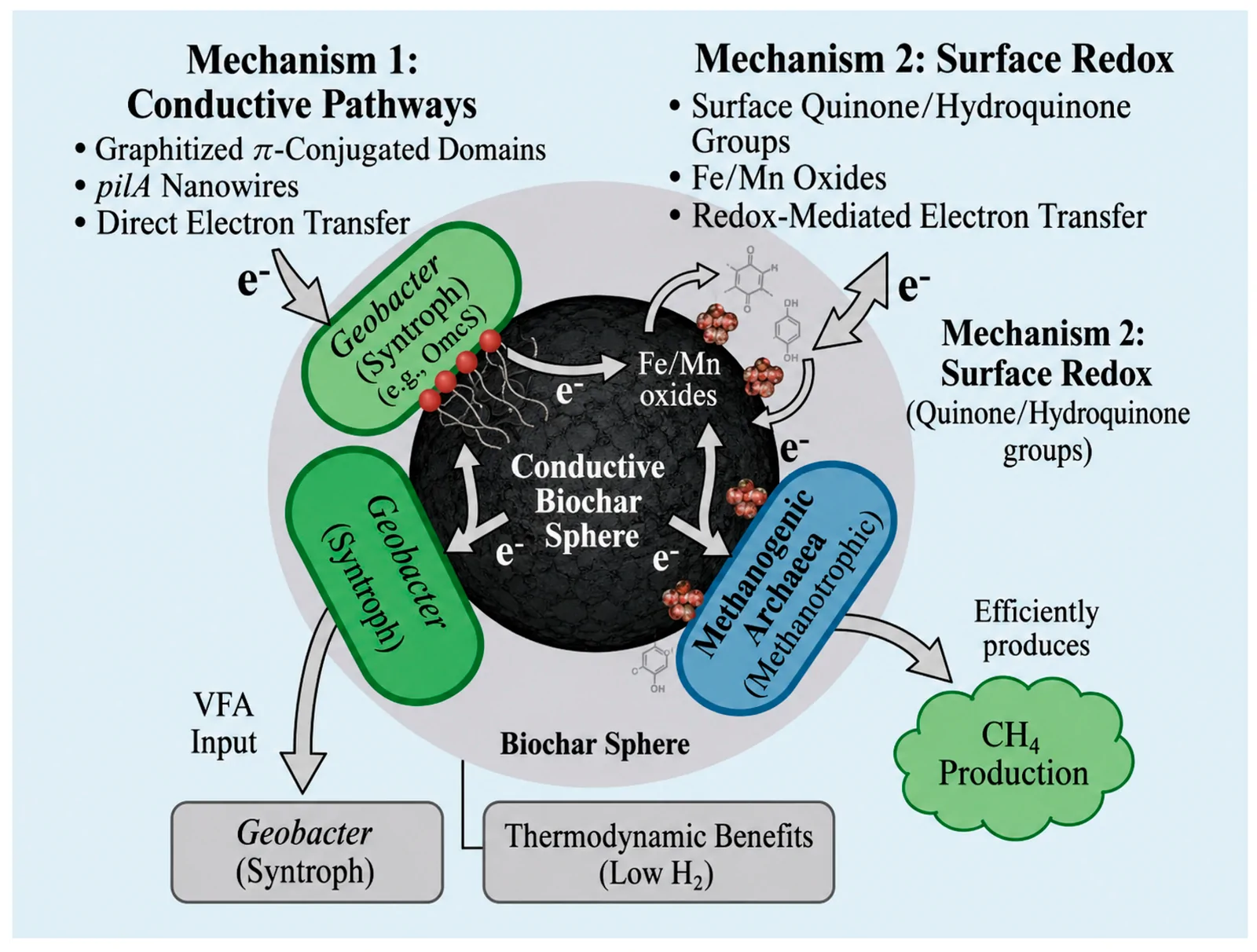
The microbiological mechanism of biochar enhancing anaerobic digestion.

Syntrophic metabolism in AD traditionally depends on indirect interspecies electron transfer (IET) pathways through the diffusion of soluble carriers like hydrogen and formate. This process is sensitive to diffusion limitations and requires extremely low hydrogen partial pressure to keep the oxidation of propionate, butyrate, and acetate thermodynamically feasible. Once hydrogen accumulates, VFA oxidation can become unfavorable, causing intermediate accumulation and process instability [93,94]. Direct interspecies electron transfer (DIET) breaks this deadlock, as shown in Figure 5. It allows electrons to flow directly from syntrophs to methanogenic archaea through conductive pili, outer-membrane cytochromes, conductive particles, or redox-active surfaces [16,95,96,97].

Biochar can promote electron transfer through two main pathways. The first is conductive bridging. Graphitic and π-conjugated carbon structures were constructed under high-temperature pyrolysis or nitrogen doping conditions. This structure can reduce charge-transfer resistance and provide conductive connections between microorganisms [15,98,99]. This function acts similarly to a metallic wire, such as granular activated carbon, magnetite, or carbon cloth [86,100,101]. Therefore, the conductivity-related advantage of biochar should not be considered unique. Its practical value lies in the combination of conductivity with pore structure, surface functional groups, microbial habitat function, and waste-derived availability. The second is the electron shuttling pathway. Biochar surfaces are often functionalized with redox-active moieties (e.g., quinone/hydroquinone groups) or transition metals (Fe/Mn oxides). These groups can mediate electron shuttling or electron hopping between the biochar and microbe interface [102]. Recent evidence suggests that biochar-enhanced syntrophic acetate oxidation may involve electron hopping through cytochrome c and riboflavin-like redox units in extracellular polymers, with biochar serving as an interfacial electron acceptor and facilitator [51,103]. This finding indicates that biochar-mediated electron transfer cannot be explained by bulk conductivity alone.

Biochar-mediated electron transfer broadens the thermodynamically feasible ΔG′ window for VFA oxidation. Here, the ΔG′ window refers to the range of actual Gibbs free energy under reactor conditions in which syntrophic reactions such as propionate, butyrate, or acetate oxidation can proceed [70,104]. These reactions are only favorable when electrons or reducing equivalents are rapidly consumed by partner methanogens. By lowering electron-transfer resistance and improving contact between syntrophic partners, biochar may help maintain more favorable ΔG′ conditions and accelerate VFA turnover under high organic loading. Microbial and molecular evidence is generally consistent with this interpretation. Systems amended with conductive biochar typically enrich the abundance of electroactive syntrophs (i.e., *Geobacter* and *Shewanella*) and their methanogenic partners (i.e., *Methanosarcina* and *Methanosaeta*) [17,105]. Multi-omics and qPCR analyses generally observe the upregulation of genes encoding conductive pili, outer-membrane cytochromes (omcS/omcZ) and other genes related to extracellular electron-transfer mcrA [36,96,106]. Additionally, magnetic or iron-modified biochar may further increase cytochrome c activity, extracellular polymeric substance electroactivity, and ATP production [68,107,108]. However, such evidence remains largely insufficient unless combined with electrochemical measurements, isotope tracing, or in situ interfacial characterization.

Therefore, future studies should decouple DIET from other biochar functions, including pH buffering, inhibitor adsorption, nutrient release, and microbial immobilization. A more reliable verification strategy would be to combine reactor kinetics, electrochemical impedance spectroscopy, cyclic voltammetry, electron exchange capacity, conductive atomic force microscopy, scanning electrochemical microscopy, microbial omics, and isotope-labeled substrate conversion. This integrated approach would help clarify whether biochar enhances AD through true DIET, redox shuttling, electron hopping, or indirect improvement of microbial conditions.

## 4. Challenges and Future Perspectives

Although biochar has shown considerable potential for improving anaerobic digestion (AD), its standardized and large-scale application remains limited by material heterogeneity, uncertain mechanism attribution, insufficient long-term validation, and incomplete sustainability assessment. These challenges are not independent. In many cases, the same biochar property that improves one function may weaken another. For example, a large surface area may enhance adsorption but may also reduce substrate or nutrient availability; strong chemical modification may improve redox activity but introduce residual reagents or metal leaching; and fine particles may improve dispersion but complicate material recovery. Therefore, future studies should move from empirical biochar addition toward application-oriented design, mechanism verification, and engineering validation.

### 4.1. Challenges

#### 4.1.1. Material Heterogeneity and Lack of AD-Specific Standards

Biochar performance in AD is governed by a coupled property set, including surface area, porosity, ash mineralogy, alkalinity, CEC, surface oxygenated functional groups, and electron-exchange capacity. These properties are strongly determined by feedstock composition, pyrolysis temperature, residence time, activation method, and post-modification [70,71,72]. However, biochar is often still reported as a generic additive rather than an AD-grade material with defined functional indicators.

Different AD mechanisms require different material descriptors. When pH buffering is the dominant function, ash mineralogy, carbonate content, alkalinity reserve and CEC are more relevant than conductivity [70,71,72]. When inhibition mitigation is the target, accessible pore structure, surface acid–base chemistry, hydrophobicity, and ion-exchange capacity become more important. When electroactive syntrophy or DIET is proposed, electron exchange capacity, redox-active moieties, surface cytochrome interactions, and interfacial charge-transfer resistance should be evaluated together with bulk conductivity. Recent studies have also shown that pore structure and microbial accessibility may be more important than a specific surface area alone, and that surface functional groups can affect different AD stages in distinct ways [10,47]. Therefore, the absence of standardized AD-oriented characterization makes it difficult to compare results across studies or predict performance in specific reactors.

#### 4.1.2. Mechanistic Attribution Remains Largely Indirect

The improvement of AD performance by biochar has been attributed to pH buffering [70,71,72], adsorption and ion exchange of NH_4_^+^, VFAs, H_2_S, and metals [87], microbial immobilization [80], and DIET mediated by conductive matrices or redox shuttles [95,98,99,109]. However, their relative contributions are seldom quantified under realistic loading. This creates uncertainty in explaining why biochar works in one system but not in another.

DIET is a typical example. Many studies infer DIET from methane enhancement, enrichment of electroactive microorganisms, or upregulation of genes related to pili, cytochromes, and methanogenesis. These indicators are useful, but they are not sufficient to prove direct electron transfer. Recent studies suggest that biochar-mediated electron transfer may also involve electron hopping through cytochrome c and riboflavin-like redox units at the biochar–microbe interface, while some methane enhancement may originate from non-DIET effects such as residual biodegradable carbon in low-temperature biochar [26,38]. Therefore, future mechanism studies should combine process kinetics, electrochemical impedance spectroscopy, cyclic voltammetry, electron exchange capacity, conductive atomic force microscopy, scanning electrochemical microscopy, isotope tracing, and multi-omics analysis. Without such integrated evidence, it is difficult to separate DIET from H_2_/formate-mediated interspecies electron transfer, pH buffering, adsorption, and microbial immobilization.

#### 4.1.3. Engineering Constraints, Recovery, and Environmental Safety

Most existing evidence is still derived from batch tests or short semi-continuous experiments using simplified substrates. In full-scale or long-term continuous systems, biochar behavior is affected by particle settling, floating, aggregation, biofilm overgrowth, pore blockage, mineral precipitation, and surface aging. These changes may alter adsorption capacity, electron transfer activity, and microbial colonization over time. Bench-scale evidence further shows that the spatial distribution, particle size, and mixing intensity of biochar can influence biogas production and reactor stability, while smaller particles may improve dispersion but complicate recovery and possibly intensify microbial over-colonization [51].

Material recovery is another unresolved issue. If powdered biochar remains in digestate, it may affect sludge dewatering, composting, land application, or downstream pyrolysis. Magnetic biochar, granular biochar, packed-bed carriers, moving-bed systems, and biochar-based composite carriers may improve retention and recovery, but their long-term stability and cost-effectiveness still need validation. Recent studies using magnetic biochar and digestate bioaugmentation demonstrated rapid recovery of collapsed AD systems, suggesting the potential of recoverable or carrier-type biochar for engineering applications [91]. However, magnetic modification may introduce additional costs, metal leaching risk, and preparation complexity. For aqueous applications, effective separation strategies are essential to recover biochar after use. Depending on particle characteristics, recovery can be achieved through filtration, sedimentation, centrifugation, membrane separation, or magnetic separation for magnetically modified biochars. After recovery, residual microbial biomass and surface-bound organic compounds can be removed or controlled through washing, mild thermal regeneration, or other low-energy regeneration approaches. Compared with direct incineration, regeneration and repeated utilization of spent biochar are more favorable for maintaining carbon sequestration benefits and reducing life-cycle carbon emissions.

The environmental safety of biochar should also be considered throughout its life cycle. High-performance modification routes may rely on strong acids, strong alkalis, oxidants, reductants, or metal precursors, creating upstream environmental, health, and safety burdens [63]. Excessive metal loading can block pores, suppress microbial activity, and increase leaching risk. In addition, biochar may contain or generate polycyclic aromatic hydrocarbons, heavy metals, and persistent free radicals depending on feedstock and production conditions [110]. Therefore, large-scale application requires systematic evaluation of leachability, digestate safety, ecotoxicity, greenhouse gas emissions, and techno-economic feasibility.

### 4.2. Future Perspectives

#### 4.2.1. Establish AD-Grade Biochar Standards and Green Manufacturing Routes

Future studies should define biochar as an AD-grade functional material rather than a generic sorbent. A minimum characterization dataset should include surface area and pore-size distribution, ash mineralogy, titratable alkalinity, cation exchange capacity, surface functional groups, electrical conductivity, electron exchange capacity, particle size, density, leachability of metals, PAHs, and residual reagents. These indicators should be reported together with substrate type, inoculum source, reactor configuration, organic loading rate, and operating temperature.

Biochar design should be guided by the dominant AD bottleneck. For acidification-prone systems, mineral-rich and alkaline biochar with sufficient buffering capacity may be preferred. For ammonia, sulfide, or antibiotic inhibition, accessible pores, cation exchange capacity, and surface functional groups should be prioritized. For slow syntrophic metabolism, redox-active or conductive biochar may be more suitable. For engineering applications, magnetic, granular, or immobilized biochar may be more practical than fine powdered biochar. At the manufacturing level, green modification strategies should be prioritized, including inherent feedstock doping, use of Fe-rich sludge or digestate, mild activation, and one-step pyrolysis-based functionalization. These approaches may reduce chemical consumption and secondary pollution while maintaining functional performance.

#### 4.2.2. Strengthen Mechanism Verification and Long-Term Continuous Validation

Future studies should move beyond short-term methane-yield comparisons. Long-term continuous trials of 6–12 months are needed for major substrates such as sewage sludge, food waste, livestock manure, agricultural residues, and high-strength industrial wastewater. These trials should quantify not only methane yield, but also VFA profiles, alkalinity, ammonia, sulfide, microbial activity, electron transfer indicators, biochar retention, surface aging, and digestate quality. The stability of biochar functions under repeated loading shocks, pH fluctuation, salinity stress, antibiotic exposure, and ammonia inhibition should also be tested.

To support practical adoption, future research should integrate life-cycle assessment, techno-economic analysis, carbon accounting, and risk assessment. Key questions include whether biochar preparation energy can be offset by methane enhancement, whether carbon credits can compensate for additional processing costs, whether modified biochar is safer than conventional additives, and whether biochar-laden digestate is suitable for agricultural use. Only when process performance, environmental safety, and economic feasibility are evaluated together can biochar-assisted AD be translated from laboratory intensification to scalable waste valorization.

## 5. Conclusions

In summary, biochar represents a promising additive for intensifying anaerobic digestion (AD) by enhancing process stability and methane recovery through physicochemical buffering and direct interspecies electron transfer (DIET) pathways. To achieve industrial scalability, future efforts should prioritize material standardization, mechanistic decoupling, and long-term pilot-scale validation. Crucially, the role of biochar extends beyond methane production by enabling AD to be upgraded into a high-value biorefinery platform for hydrogen and medium-chain fatty acid production through regulation of carbon and electron fluxes.

## Figures and Tables

**Table 2:** Representative microbial and metabolic responses to biochar addition in the AD system.

Biochar Type/Modification	Substrate	Performance	Microbial/Metabolic Response	Proposed Mechanism	Representative Reference
Tubular-pore biochar	Waste-activated sludge	Increased cumulative methane yield by 17.6%	Enriched functional bacteria and methanogens, especially Romboutsia and Methanosaeta	Tubular pore structure promoted hydrolysis, microbial colonization, and potential DIET	Ref. [50]
Porous biochar	Organic substrates	Improved methane yield and process stability	Enriched syntrophic bacteria	Reduced diffusion distance for H_2_, formate, acetate	Ref. [84]
Biochar (general)	Complex organics	Enhanced substrate conversion	Promoted hydrolytic and fermentative guilds	Microhabitat formation and biomass retention	Ref. [84]
Mineral-releasing biochar	Food waste/sludge	Stable CH_4_ production under load fluctuation	Stimulated fermentative bacteria	Release of Fe, Co, Ni as enzyme cofactors	Refs. [85,86,87]
N-, Ca-, Fe-, Mn-doped biochar	Carbohydrate-rich substrates	Hydrogen yield enhanced	*Clostridium sensu stricto 1*, *C. butyricum* enriched	Selective enrichment of hydrogen-producing bacteria	Refs. [84,85,86,88]
Biochar-amended system	Organic substrates	H_2_ production accelerated	Promoted cellulolytic and fermentative bacteria	Increased cellulase and hydrogenase activities	Ref. [84]
Biochar addition	Sugars and VFAs	Shift toward acetate/ethanol pathways	Reduced competing pathways	Improved NAD+/NADH balance for proton reduction	Ref. [84]
Biochar + Fe^0^/Ni^0^ nanoparticles	Organic substrates	Synergistic increase in H_2_ yield	Enriched electron-transferring bacteria	Enhanced IET and EET within biofilms	Refs. [67,89]
Magnetic biochar	Waste-activated sludge; high-concentration organic wastewater	Formed synergistic enhancement compared with biochar and magnetite alone	Enriched Peptoclostridium, Anaerolineaceae, *Methanosarcina*, and Methanosaeta	Enhanced EPS electroactivity, cytochrome c, ATP supply, and DIET-related metabolism	Refs. [7,8]
Quinone-modified biochar	High-load corn straw	VFAs decreased by 77.38%; biogas production increased by 177.87%	Enriched Lentimicrobium, Flexilinea, Methanobacterium, and *Methanosarcina*	Quinone groups enhanced redox mediation, c-type cytochrome-related genes, and coenzyme F420 synthesis	Ref. [82]
Digestate-based biochar	High ammonia	Methane yield increased by 20.02% under ammonia stress	Enriched Firmicutes, Synergistota, Methanobacterium, and Methanomassiliicoccus	Improved ammonia adsorption, pH buffering, microbial adaptation, and hydrogenotrophic/methylotrophic methanogenesis	Ref. [23]
ZVI-modified biochar	Aquaculture wastewater AD under OTC and SMX stress	Maintained stable methane production under combined antibiotic stress	Increased resilience of *Methanothrix*-dominated methanogenic community	Reduced antibiotic bioavailability through microporous adsorption and restored IET via zero-valent iron	Ref. [83]
Cow-dung-derived biochar with inherent Fe	Swine wastewater AD under ciprofloxacin stress	Methane production increased by 386% compared with CIP-stressed control	Enriched Clostridium and *Methanothrix*	Inherent Fe and oxygen-containing groups promoted pollutant mitigation, acidogenesis, methanogenesis, and electron transfer	Ref. [82]

## Data Availability

No new data were created or analyzed in this study. Data sharing is not applicable to this article.
